# Automated antimicrobial susceptibility testing and antimicrobial resistance genotyping using Illumina and Oxford Nanopore Technologies sequencing data among *Enterobacteriaceae*

**DOI:** 10.3389/fmicb.2022.973605

**Published:** 2022-08-08

**Authors:** Rick Conzemius, Yehudit Bergman, Peter Májek, Stephan Beisken, Shawna Lewis, Emily B. Jacobs, Pranita D. Tamma, Patricia J. Simner

**Affiliations:** ^1^Ares Genetics GmbH, Vienna, Austria; ^2^Department of Pathology, Johns Hopkins University School of Medicine, Baltimore, MD, United States; ^3^Department of Pediatrics, Johns Hopkins University School of Medicine, Baltimore, MD, United States

**Keywords:** antimicrobial susceptibility testing, molecular diagnostics, carbapenem-resistant *Enterobacteriaceae*, whole-genome sequencing, nanopore sequencing, point-of-care testing, WGS-AST

## Abstract

Whole-genome sequencing (WGS) enables the molecular characterization of bacterial pathogens. We compared the accuracy of the Illumina and Oxford Nanopore Technologies (ONT) sequencing platforms for the determination of AMR classes and antimicrobial susceptibility testing (AST) among 181 clinical *Enterobacteriaceae* isolates. Sequencing reads for each isolate were uploaded to AREScloud (Ares Genetics) to determine the presence of AMR markers and the predicted WGS-AST profile. The profiles of both sequencing platforms were compared to broth microdilution (BMD) AST. Isolates were delineated by resistance to third-generation cephalosporins and carbapenems as well as the presence of AMR markers to determine clinically relevant AMR classes. The overall categorical agreement (CA) was 90% (Illumina) and 88% (ONT) across all antimicrobials, 96% for the prediction of resistance to third-generation cephalosporins for both platforms, and 94% (Illumina) and 91% (ONT) for the prediction of resistance to carbapenems. Carbapenem resistance was overestimated on ONT with a major error of 16%. Sensitivity for the detection of carbapenemases, extended-spectrum β-lactamases, and plasmid-mediated *ampC* genes was 98, 95, and 70% by ONT compared to the Illumina dataset as the reference. Our results highlight the potential of the ONT platform’s use in clinical microbiology laboratories. When combined with robust bioinformatics methods, WGS-AST predictions may be a future approach to guide effective antimicrobial decision-making.

## Introduction

The emergence of antimicrobial resistance (AMR) is recognized by leading health organizations as one of the major threats to global health. The accelerated progression of AMR requires not only the development of new therapeutics but also rapid, comprehensive, and accurate diagnostic methods to guide early and effective antimicrobial therapy (Livermore, 2004; Rochford et al., 2018; World Health Organization, 2018, 2020).

The Infectious Diseases Society of America’s treatment guidance for infections caused by multidrug-resistant Gram-negative organisms highlights the importance of understanding the mechanisms mediating AMR, as antimicrobial selection may differ by mechanism. As an example, discerning whether a bloodstream infection caused by a third-generation cephalosporin-resistant organism is mediated by extended-spectrum β-lactamase (ESBL) versus AmpC β-lactamase production is important, as the recommended therapy for the former is a carbapenem and cefepime for the latter. Similarly, understanding whether a carbapenem-resistant Enterobacterales (CRE) infection is caused by the presence of a *bla*_KPC_ gene versus a *bla*_OXA-48-like_ gene is of significance, as the former is generally effectively treated by meropenem-vaborbactam whereas the latter is not.

Whole-genome sequencing (WGS) provides the ability to identify the resistome of microorganisms (i.e., all antimicrobial resistance genes harbored) but can also be used for antimicrobial susceptibility testing (WGS-AST) and potentially guide antimicrobial decision making. We have previously shown that machine learning-based WGS-AST has several advantages over rule-based AMR gene detection for determining susceptibility or resistance to antimicrobials (Lüftinger et al., 2021a). The Oxford Nanopore Technologies (ONT) WGS platform offers rapid and easy library preparation, live readout, reduced turn-around time, lower initial investment, and per-run costs compared to standard platforms such as Illumina (Greninger et al., 2015; Jain et al., 2015; Quick et al., 2016; Schmidt et al., 2017; Charalampous et al., 2019). A significant disadvantage of ONT, however, is the higher per-base error rate, with accuracies between 90 and 99%, depending on the chemistry used, compared to Illumina with 99.9% raw read accuracy. The lower fidelity of ONT reads necessitates the use of specialized bioinformatics tools to mitigate these impacts on downstream analysis (Rang et al., 2018; Wang et al., 2021).

We investigated predicted WGS-AST and AMR gene detection of 181 clinical *Enterobacteriaceae* isolates using sequencing data acquired on both the Illumina and ONT platforms. We compared the WGS-AST profiles to phenotypic broth microdilution (BMD) AST results to determine the overall categorical agreement (CA), very major error (VME), and major error (ME) for ONT and Illumina. We further assessed the suitability of both platforms to determine clinically relevant AMR classes associated with third-generation cephalosporin resistance and carbapenem resistance by combining WGS-AST results with detected molecular markers in a decision tree.

## Materials and methods

### Isolates

A total of 181 clinical *Enterobacteriaceae* isolates recovered from patients at The Johns Hopkins Hospital (Baltimore, MD) between 2016 and 2020 were included in the current study. The included organisms were as follows: *Klebsiella pneumoniae* [n: 151], *K. quasipneumoniae* [n: 3], *Escherichia coli* [n: 14], *Enterobacter cloacae* [n: 7], *E. hormaechei* [n: 3], *E. chengduensis* [n: 1], *E. kobei* [n: 1], and *E. roggenkampii* [n: 1]. Of these, 49 isolates were carbapenem-susceptible and 132 were carbapenem-resistant. Isolates were subcultured from frozen stocks twice on tryptic soy agar with 5% sheep blood (BD Diagnostics, Sparks, MD) for 18–24 h at 37°C.

### Identification

Bacterial genus and species were determined by matrix-assisted laser desorption ionization time-of-flight mass spectrometry (MALDI-TOF MS; Bruker Daltonics Inc., Billerica, MA).

### Antimicrobial susceptibility testing

Broth microdilution AST (BMD-AST) was performed with the Sensititre GN7F and MDRGN2F (Thermo Fisher Scientific, Waltham, MA, United States) panels for Gram-negative bacteria following Clinical and Laboratory Standards Institute (CLSI) guidelines for a total of 31 antimicrobials. BMD-AST results were interpreted according to CLSI guidelines M100-S31, except fluoroquinolones were interpreted according to M100-S28 due to the restricted dilutions on the panel (CLSI, 2018, 2021). For all BMD-AST studies, quality control organisms were evaluated each day of testing.

### DNA extraction

Genomic DNA was extracted from pure cultures using the DNeasy PowerSoil Pro and DNeasy PowerBiofilm kits (QIAGEN, Hilden, Germany), following the manufacturers’ guidelines.

### Illumina sequencing

The sequencing libraries were prepared using the Nextera DNA Flex Library Preparation Kit (Illumina, San Diego, CA, United States). Library concentrations were verified using a dsDNA fluorescent dye method on the Qubit 3.0 Fluorometer (Thermo Fisher Scientific, MA, Waltham, United States). DNA fragment size and library quality were confirmed on a 4200 TapeStation system (Agilent, Santa Clara, CA, United States). Cluster generation was performed on the cBot System (Illumina, San Diego, CA United States) using the HiSeq Rapid PE Cluster v2 and HiSeq Rapid Duo cBot Sample Loading kits (Illumina, San Diego, CA United States). Libraries were sequenced on HiSeq 2500 (using the rapid run mode) and MiSeq devices (Illumina, San Diego, CA, United States) at 7 pmol with HiSeq Rapid SBS Kit v2 (2 × 250 bp, 500-cycle kit), MiSeq Reagent Kit v2 (2 × 150 bp, 300-cycle kit), or MiSeq Reagent Kit v3 (2 × 300 bp, 600-cycle kit) chemistries, respectively.

### Oxford Nanopore Technologies sequencing

Long-read genomic sequencing was performed using the third generation Oxford Nanopore MinION Mk1B and GridION X5 (Oxford, United Kingdom) sequencing instruments. Each Nanopore sequencing library was prepared using the 1D ligation kit (SQK-LSK108, Oxford Nanopore Technologies) and sequenced on R9.4 flow cells (FLO-MIN106). High-accuracy live base calling was done with Guppy 4.3+ (Oxford Nanopore, Oxford, United Kingdom) as released with the MinKNOW (Oxford Nanopore, Oxford, United Kingdom) software.

### Data processing

Sequencing reads were uploaded to AREScloud (Ares Genetics) for genome assembly, quality control, identification, sequence typing, AMR marker detection, and WGS-AST (Ares Genetics GmbH, 2021). For Illumina sequencing, isolates were evaluated using FastQC v0.11.5 and fastq-stats v1.01 (Aronesty, 2011; Andrews, 2016). Datasets exceeding 4,000,000 reads were randomly subsampled using seqtk v1.2-r94 (Li, 2016). Adapter removal and trimming of low-quality paired-end reads were done using Trimmomatic v0.39 (Bolger et al., 2014). Reads were *de novo* assembled with SPAdes v3.15.25 (Prjibelski et al., 2020). ONT sequencing quality was evaluated using NanoPlot v1.39.0 and low-quality reads were removed with NanoFilt v.2.8 at a quality threshold of 7 (de Coster et al., 2018). Datasets were subsampled to 600 Mb with rasusa v0.6.0, *de novo* assembled with Canu v2.1.1, and iteratively polished with Racon v1.5 (Koren et al., 2017; Vaser et al., 2017; Hall, 2021). For both sequencing platforms, assembly quality and genome completeness were determined using Quast v5.0.2 and BUSCO v5.2.2 (Mikheenko et al., 2018; Manni et al., 2021). Insert size for Illumina datasets was calculated using CollectInsertSizeMetrics v2.25.2 from Picard Tools (Broad institute, 2021). *De novo* assemblies were annotated with Prokka v1.14.1 and ribosomal RNA genes were identified using Barrnap v0.9 (Seemann, 2014, 2018). The genome annotations and ribosomal RNA genes were used for assembly quality evaluation; the Prokka features also constitute part of the input feature space for the WGS-AST machine learning models. Microbial identification was determined with Kraken v2.0.9-beta using the MiniKraken PlusPF-8 database from 2021/05/17 (Wood and Salzberg, 2014). Genome coverage was determined using bwa mem v0.7.17-r1188 and bedtools v2.29.0 (Quinlan and Hall, 2010; Li, 2013). Resistance genes were determined with DIAMOND v1.0.11 *via* sequence alignment of six-frame translated genome assemblies against ARESdb with a minimum query coverage of 60% and a minimum identity of 90% for Illumina-derived assemblies and a minimum identity of 70% for ONT-derived assemblies (Buchfink et al., 2015; Ferreira et al., 2020). WGS-AST results were generated by susceptibility/resistance (S/R) stacked classification models trained per species-antimicrobial pair on ARESdb (Ferreira et al., 2020). The model stacks combine extreme gradient boosting, elastic net regularized logistic regression (ENLR), and set covering machine models as well as rule-based post-processing routines. ARESdb models were trained on features derived from sequence motifs and variants (Lüftinger et al., 2021b; Májek et al., 2021). Ertapenem was used as a proxy for the prediction of resistance to carbapenems and was optimized from previous publications, to recognize non-CP CREs (Májek et al., 2021) by recognizing changes in the *ompK35*, *ompK36,* and homologs (Doumith et al., 2009; Sugawara et al., 2016). WGS-AST was run for 60 species-antimicrobial pairs listed in Supplementary Table 1. Discrepant species identifications between the Illumina and ONT platforms were confirmed by comparing the assemblies to the reference genomes of the suspected species using FastANI (Jain et al., 2018). Data processing on AREScloud took less than 2 h for Illumina data and up to 5 h for ONT data; the application scales horizontally, i.e., samples are processed in parallel on multiple instances without decrease in computing power.

### AMR class definition

**Figure 1:**
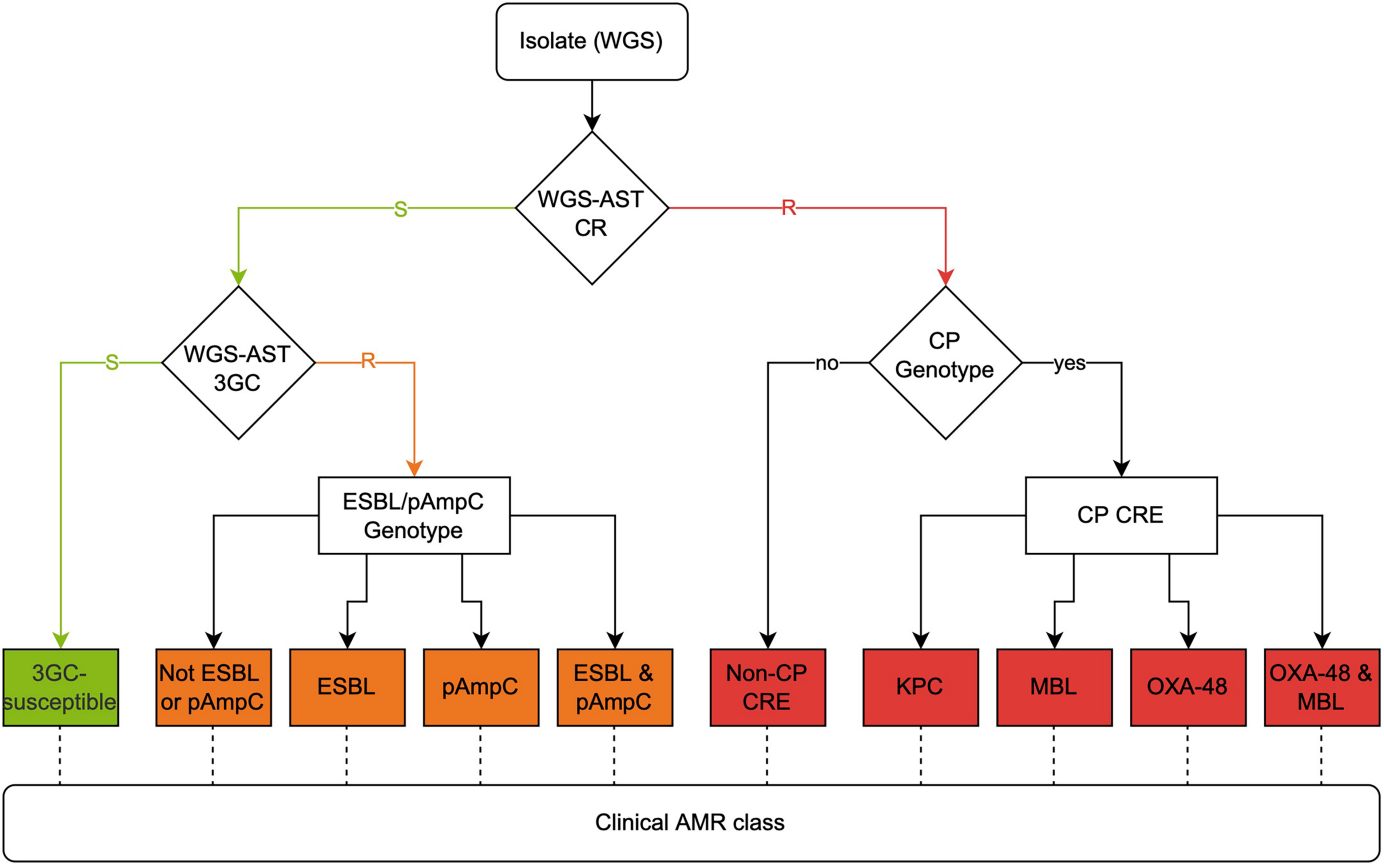
Clinical AMR class decision workflow. WGS-AST: whole-genome sequencing antimicrobial susceptibility testing; CR: carbapenem resistance; CRE: carbapenem-resistant Enterobacterales; 3GC: third-generation cephalosporin resistance, CP: carbapenemase; ESBL: extended-spectrum β-lactamase; pAmpC: plasmid AmpC; MBL: metallo-β-lactamase.

Third-generation cephalosporin (3GC) resistance was defined by resistance to ceftriaxone; carbapenem resistance (CR) was defined by resistance to ertapenem. Molecular genotypes (ESBL, plasmid-mediated AmpC beta-lactamase (pAmpC), *Klebsiella pneumoniae* carbapenemase (KPC), metallo-β-lactamase [MBL], OXA-48-like) and WGS-AST results were combined to define AMR classes, as outlined in the decision tree in Figure 1. Briefly, 3GC-resistant, carbapenem-susceptible organisms were delineated by pAmpC and ESBL markers. CP-CREs were characterized by KPC, MBL, and OXA-48-like marker genes. Non-CP CREs were defined by resistance to ertapenem by WGS-AST and the absence of carbapenemase genes. WGS-AST performance and concordance of AMR classes were finally compared between platforms.

### Statistical evaluation

Categorical agreement (CA), very major error (VME), and major error (ME) were assessed for WGS-AST. Resistance (R) was considered a positive outcome and susceptibility (S) a negative outcome. WGS-AST S/R results were classified either as true positives (TP), false positives (FP), true negatives (TN), or false negatives (FN), in comparison to BMD-AST results. Intrinsically resistant (IR) isolates, and isolates with an intermediate (I) phenotype, were treated as resistant. Categorical agreement (accuracy) of S/R designations was compared between WGS-AST and BMD-AST; very major errors (VME) are false-susceptible results (FNR, false-negative rate) by WGS-AST, and major errors (ME) are false-resistant results (FPR, false-positive rate) by WGS-AST. Species-antimicrobial pairs with less than 10 samples were removed: ampicillin (n: 9), ampicillin-sulbactam (n: 9), and minocycline (n: 6). Data handling and analysis was carried out in a Python environment using the Pandas and NumPy libraries. Confusion matrices were generated using the scikit-learn library and statistical tests were calculated using the SciPy library.

## Results

### Phenotypic antimicrobial susceptibility testing results

Phenotypic antimicrobial susceptibility testing results are summarized in Supplementary Table 2. Of the 181 isolates, 135 were not susceptible (NS; i.e., intermediate or resistant) to at least one carbapenem (ertapenem: 72.9%, doripenem: 55.6%; imipenem: 61.1%: meropenem: 54.5%); 144 isolates were NS to 3GCs, while 136 isolates were NS to fourth-generation cephalosporins. Non-susceptibility to newer β-lactam/β-lactamase inhibitor combinations was low (imipenem-relebactam: 10.5%, meropenem-vaborbactam: 10.6%, ceftazidime-avibactam: 6.8%). NS to non-β-lactam agents ranged among isolates as follows: 8.3–51.7% for aminoglycosides, 64.1–88.9% for fluoroquinolones, 38.0% for tetracycline, and 49.4% for sulfamethoxazole-trimethoprim.

### Sequencing and assembly

ONT assemblies had lower BUSCO completeness scores of 42.59% ± 23.59% compared to 98.57% ± 0.66% on the Illumina platform. ONT assemblies were less fragmented as determined by BUSCO (30.48% ± 12.65%) compared to Illumina assemblies (0.02% ± 0.15%); BUSCO missing scores were worse for ONT (26.92% ± 11.36%) than for Illumina data (1.40% ± 0.59%), highlighting the higher per-base error rates on the ONT platform. All comparisons were significant (two-tailed paired *t*-test, *p* < 0.0001).

### Identification

The genus of all 181 isolates (100%) was correctly identified for ONT and Illumina isolates. Species-level concordance was 97.24%; 5 *Enterobacter* isolates were identified on the ONT assemblies as *E. hormaechei* and on the Illumina assemblies as *E. cloacae*. The discrepant isolates were confirmed belonging to the *E. hormaechei* species using the average nucleotide identity (ANI) method at a cutoff of 95% (Supplementary Table 3; Jain et al., 2018).

### AMR markers

The number of uniquely identified AMR markers was on average 19.9% higher on the ONT dataset than on the Illumina dataset (Supplementary Table 4). The higher number of retrieved markers on ONT assemblies was due to the lower identity threshold used by DIAMOND v1.0.11 for sequence alignment of AMR markers to ARESdb. The threshold was optimized for ONT data to retrieve all relevant markers needed to assign AMR classes. Due to the higher fragmentation of ONT assemblies and lower base calling fidelity, incorrect alleles were assigned more often on ONT assemblies (two-tailed paired *t*-test, *p* < 0.001).

With the Illumina data as the reference, the sensitivity of AMR markers in the ONT dataset was investigated. The sensitivity of carbapenemases, ESBLs, and pAmpCs was slightly lower on ONT compared to Illumina at 98, 95, and 70%, respectively, with specificities of 100, 70, and 91%, respectively. Among the carbapenemases, accuracy was 98, 100, and 99% for KPCs, MBLs, and OXA-48-like AMR marker genes, respectively. The sensitivity was lowest for pAmpCs (70%), and specificity was lowest for ESBLs (70%). Across all 6 marker classes, the specificity of ONT was 100% for carbapenemases, KPCs, metallo-β-lactamases, and OXA-48-like markers. The lowered sensitivity of ONT led to a high false-negative rate (FNR) in some cases, e.g., 30% for pAmpCs (Table 1).

**Table 1:** Performance metrics of the AMR marker identification on ONT data, which is compared to the markers identified on the Illumina data (reference).

Marker class	Accuracy	Sensitivity	Specificity	FNR	FPR	TP	FP	FN	TN	*n*
ESBL	78%	95%	70%	5%	30%	79	29	4	69	181
pAmpC	96%	70%	91%	30%	9%	16	0	7	158	181
CP	99%	98%	100%	2%	0%	99	0	2	80	181
KPC	98%	96%	100%	4%	0%	74	0	3	104	181
MBL	100%	100%	100%	0%	0%	15	0	0	166	181
OXA-48	99%	93%	100%	7%	0%	13	0	1	167	181

CA: Categorical agreement, FNR: False-negative rate, FPR: False-positive rate, TP: True positive, FP: False positive, FN: False negative, TN: True negative, and n: number of evaluated samples.

### WGS-AST

Overall, ONT data performed slightly worse than Illumina sequencing data regarding WGS-AST (Table 2), but the difference was not significant (*χ*^2^ test on the vectorized confusion matrices, *p* > 0.05). Categorical agreement (CA) was 88% for ONT and 90% for Illumina, overall ME was higher on the ONT platform with 13% compared to 11% on the Illumina platform, and overall VME was also slightly higher at 11% on ONT compared to 10% on the Illumina platform. On the ONT platform, 25 species-antimicrobial models had a CA between 90 and 100%, 23 between 80 and 90%, and 12 below 80%; on the Illumina platform, 34, 18, and 8 species-antimicrobial models had CAs of 90–100%, 80–90%, and below 80%, respectively. Grouped by antimicrobial, on the ONT platform, 10 species-antimicrobial models had a CA between 90–100%, 11 between 80–90%, and 1 below 80%; on the Illumina platform, 13, 9, and zero species-antimicrobial models had CAs of 90–100%, 80–90%, and below 80%, respectively (Table 3; Supplementary Table 5). Grouped by organism, CA was highest for *Klebsiella* spp. with 89% on the ONT platform and 90% on the Illumina platform. *Enterobacter* reached a CA of 83% on the ONT platform and *Escherichia* of 82% on the ONT platform. *Escherichia* reached a CA of 87% and *Enterobacter* a CA of 85% on the Illumina platform (Supplementary Table 6). Here, two effects were observed: *Escherichia* and *Enterobacter* models had lower CAs than *Klebsiella* models and ONT had a lower CA compared to Illumina as described above. While *Klebsiella* had the highest CA, it also had a higher VME of 11% on both platforms than *Enterobacter* on ONT (6%) and Illumina (8%) platforms, and *Escherichia* on the Illumina platform (5%) but not on the ONT platform (18%). MEs of *Klebsiella* (12% vs. 9%) and *Enterobacter* models (27% vs. 22%) were higher on the ONT platform compared to the Illumina, while *Escherichia* models (19% vs. 32%) had lower MEs on the ONT platform compared to Illumina.

**Table 2:** Overall performance of the WGS-AST models across all antimicrobials, broken down by sequencing platform.

Platform	CA	VME	ME	TP	FP	FN	TN	*n*
Illumina	90%	10%	11%	1,646	161	178	1,315	3,300
ONT	88%	11%	13%	1,619	194	205	1,282	3,300

CA: Categorical agreement, VME: Very major error, ME: Major error, TP: True positive, FP: False positive, FN: False negative, TN: True negative, and *n*: number of evaluated species-antimicrobial pairs.

**Table 3:** Comparison of the performance metrics of WGS-AST on Illumina and ONT assemblies, by antimicrobial.

Antimicrobial	CA		VME		ME		*n*
	Illumina	ONT	Illumina	ONT	Illumina	ONT	
Amikacin	80%	83%	23%	20%	19%	16%	163
Aztreonam	91%	92%	9%	6%	12%	14%	180
Cefazolin	84%	84%	22%	20%	0%	6%	68
Cefepime	85%	83%	16%	21%	12%	7%	173
Cefotaxime	96%	95%	4%	4%	0%	10%	107
Ceftazidime	89%	93%	10%	5%	14%	14%	181
Ceftazidime-avibactam	97%	96%	6%	35%	3%	1%	175
Ceftriaxone	96%	96%	4%	2%	3%	7%	79
Ciprofloxacin	95%	88%	3%	12%	10%	12%	181
Doripenem	84%	83%	22%	22%	8%	11%	164
Ertapenem	94%	91%	7%	6%	4%	16%	181
Gentamicin	93%	88%	8%	8%	6%	15%	171
Imipenem	84%	79%	6%	5%	31%	44%	94
Imipenem-relebactam	97%	97%	21%	21%	1%	1%	91
Levofloxacin	90%	85%	9%	14%	11%	18%	181
Meropenem	84%	80%	3%	5%	31%	37%	90
Meropenem-vaborbactam	97%	95%	21%	36%	1%	1%	91
Piperacillin-tazobactam	90%	84%	11%	21%	7%	2%	168
Sulfamethoxazole-trimethoprim	80%	80%	11%	8%	30%	33%	167
Tetracycline	81%	82%	30%	30%	12%	10%	79
Ticarcillin-clavulanic acid	92%	95%	9%	5%	0%	0%	101
Tobramycin	94%	92%	4%	6%	7%	10%	173

CA: Categorical agreement, VME: Very major error, ME: Major error, *n*: number of evaluated species-antimicrobial pairs.

The difference in CA between individual antimicrobials spanned from a 7% lower CA to a 4% higher CA for ONT compared to Illumina sequencing data. Lower performance on ONT sequencing data was, e.g., observed in ciprofloxacin (−7%), levofloxacin (−6%), piperacillin-tazobactam (−7%), and gentamicin (−5%), while better performance of ONT was observed in, e.g., aztreonam (+2%), ticarcillin-clavulanic acid (+3%), amikacin (+3%), and ceftazidime (+4%; Table 3).

### WGS-AST models for AMR classes

Categorical agreement for ceftriaxone, which was used as a proxy for the prediction of resistance to third-generation cephalosporins, was 96% on both platforms, with ONT performing better with a lower VME (2% vs. 4%) but higher ME (7% vs. 3%).

Ertapenem—used to indicate carbapenem resistance—reached a CA of 91% on the ONT platform and 94% on the Illumina platform (*χ*^2^ test, *p* < 0.001 as tested on the vectorized confusion matrices). VMEs were comparable on both platforms (7% on ONT and 6% on Illumina), but MEs were higher on the ONT platform (16% vs. 4%; Table 3; Supplementary Table 7). On the ONT platform, MEs of the *E. cloacae* and VMEs of the *E. coli* model, and on the Illumina platform, MEs in the *Enterobacter* and *E. coli* subsets model were noticeable. On both platforms, the *Klebsiella* model performs with a better trade-off between ME and VME (Supplementary Table 5).

Ertapenem WGS-AST models reached 95% CA for CP-CREs (defined by ertapenem BMD-AST and the presence of a carbapenemase gene in the Illumina genome assembly) for data acquired on the ONT platform and 100% for data acquired on the Illumina platform, and 88% CA in the non-CP CRE dataset for ONT and 76% for Illumina platforms. Among ertapenem-susceptible isolates, CA on ONT data was lower (84%) compared to Illumina (96%), due to 8 samples being flagged positive. VMEs of ertapenem among non-CP CREs were 12% on ONT and 24% on Illumina (Supplementary Table 8).

### Combination of WGS-AST and molecular markers for AMR classes

**Figure 2:**
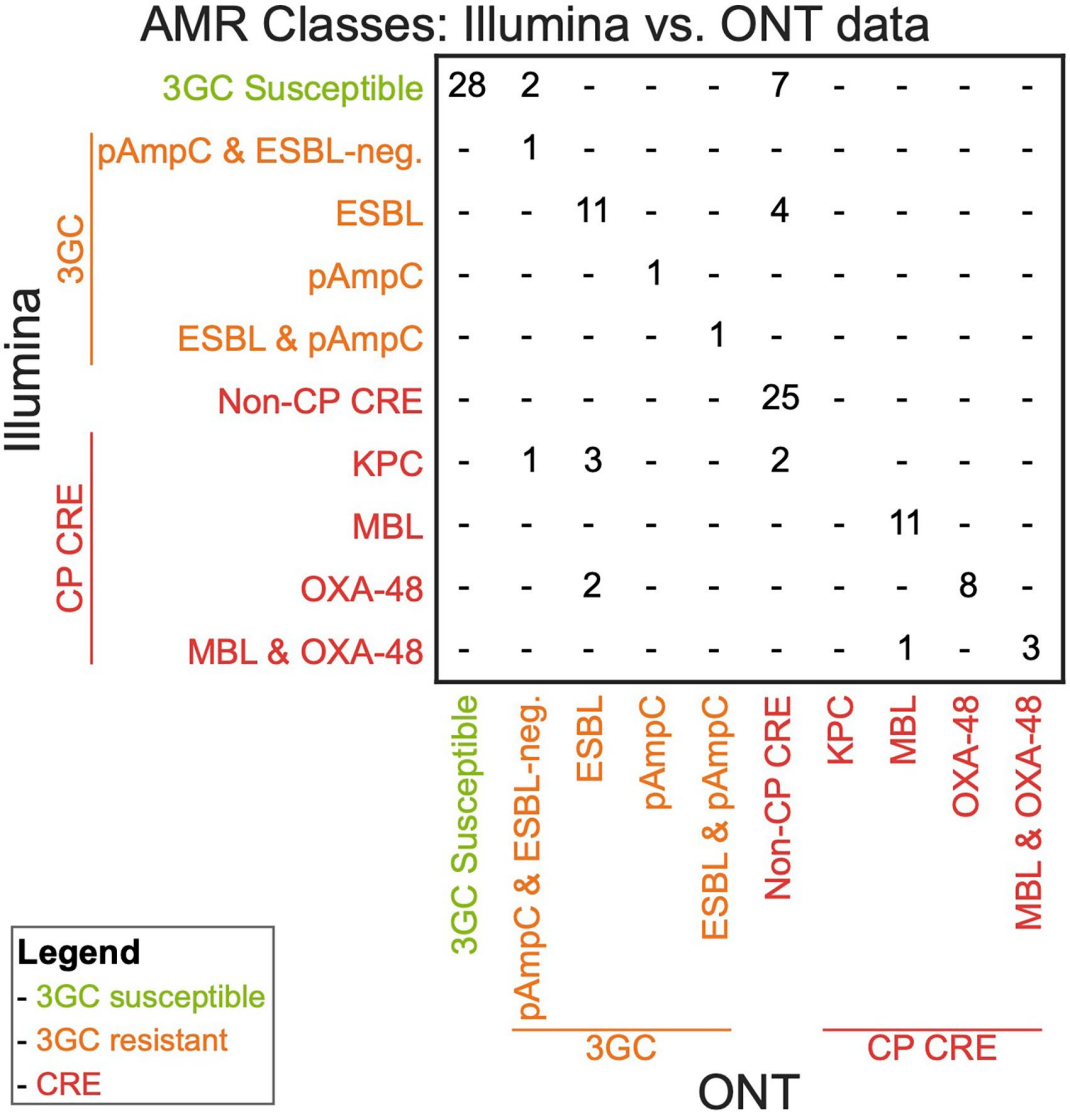
Confusion matrix of AMR classes (3GC susceptible, 3GC resistant delineated by ESBL and pAmpC, non-CP CRE, and CP CRE delineated by KPC, metallo-β-lactamase, and OXA-48) derived from Illumina and ONT WGS-AST combined with AMR marker detection.

We combined WGS-AST with the identified AMR genotypes, as outlined in Figure 1 in a decision workflow (Table 4; Supplementary Table 9). An isolate was first categorized as carbapenem-resistant or carbapenem-susceptible. Carbapenem-susceptible isolates were further classified as resistant or susceptible to 3GCs based on the ceftriaxone resistance. Error rates, as discussed in the previous section, apply to both carbapenem and 3GC resistance results. The accuracy between AMR classes predicted by ONT data and AMR classes predicted by Illumina data was 87.8% (Figure 2). For the distinction of CP CREs and non-CP CREs from WGS-AST data, data acquired on the Illumina platform was used for the detection of CP genes as the reference. Mainly, non-CP CREs were overpredicted on ONT data due to missed carbapenemase gene detection in the dataset while resistance to ertapenem was correctly predicted.

**Table 4:** Breakdown of the isolates by AMR class and sequencing platform at the decision gateways.

Group Level 1			Group Level 2			Group Level 3		
	Illumina	ONT		Illumina	ONT		Illumina	ONT
Carbapenem Susceptible	55	50	3GC Susceptible	37	28	3GC-susceptible	37	28
			3GC Resistant	18	22	Not ESBL or pAmpC	1	4
						ESBL	15	16
						pAmpC	1	1
						ESBL & pAmpC	1	1
Carbapenem Resistant	126	131	Non-CP CRE	25	38	Non-CP CRE	25	38
			CP CRE	101	93	KPC	76	70
						MBL	11	12
						OXA-48	10	8
						OXA-48 & MBL	4	3

## Discussion

Overall, this work demonstrated that the combination of WGS-AST and molecular identification of AMR markers through WGS can classify isolates into clinically relevant AMR classes regardless of sequencing platform. Second, WGS-AST on AREScloud using ONT sequencing data was comparable to Illumina sequencing data. Overall categorical agreement across all antimicrobials was 88% (ONT) and 90% (Illumina), 96% CA by both platforms for third-generation cephalosporin resistance, and 91% (ONT) and 94% (Illumina) for carbapenem resistance. VMEs (11% vs. 10%) and MEs (13% vs. 11%) were slightly higher on the ONT platform. The accuracy (89% vs. 90%) of WGS-AST predictions was slightly lower on ONT assemblies compared to Illumina. This difference was likely due to significantly higher fragmentation and higher base calling error rate of the ONT data, translating to an overall difference of 2% *CA.* Similarly, for carbapenem resistance, there were also differences in VMEs and MEs observed between the two sequencing platforms. The VMEs and MEs on the ONT platform were 7% and 16%, whereas the VMEs and MEs of the Illumina platform were 6% and 4%. The higher ME with ONT might have been due to the higher per-base error rate leading to the interpretation of random sequencing errors as SNPs in the *ompK* genes; however, since the data acquisition, ONT released new base calling models and kit chemistries allowing more complete, less fragmented assemblies, with a lower error rate, potentially lowering the ME had the analysis been repeated with the updated chemistries and base caller.

A total of 22 antimicrobials were tested for the *Enterobacteriaceae*: All antimicrobials for which WGS-AST results were predicted reached CAs above 80% and up to 97% across all species on both sequencing platforms compared to BMD-AST, except for imipenem at 79% on the ONT platform. Of the 60 individual species-antimicrobial WGS-AST models, 8 (Illumina) and 11 (ONT) models had CAs below 80%. All species-antimicrobial pairs below 80% belonged to the *Escherichia* (n: 14) or *Enterobacter* (n: 13) genera, where sample sizes were significantly lower than for, e.g., *K. pneumoniae*, hampering statistical evaluation. Thirty-three and 25 (out of 60) species-antimicrobial pairs reached CAs above 90% on the Illumina and ONT platforms, respectively (13 and 10 for *Klebsiella* on Illumina and ONT, respectively). Despite WGS-AST models of antimicrobials reaching the 90% CA threshold as defined by the FDA for the clearance of phenotypic AST devices, MEs and VMEs were outside of acceptable limits of 3% and 1.5%, respectively (Food and Drug Administration, 2009). On-going improvements of sequencing chemistries and further refinement of WGS-AST models as more data become available will likely reduce error rates to acceptable limits within the next years.

A possible mitigation strategy to deal with high error rates could be the introduction of areas of technical uncertainty (ATU), following the lead of EUCAST for phenotypic AST (The European Committee on Antimicrobial Susceptibility Testing, 2019). ATUs would help manage methodological and technical variability as well as variations in interpretation. For example, individual WGS-AST results could be annotated with a confidence level; results within an ATU could then be flagged or excluded from the report if other antimicrobials are available for treatment depending on the clinical scenario.

Regarding AMR marker retrieval, ONT was comparable to Illumina regarding accuracy (98–100%), sensitivity (93–100%), and specificity (100%) for carbapenemases, KPCs, MBLs, and OXA-48-like ß-lactamases. For pAmpCs, the sensitivity was lower at 70% due to a high number of false negatives. The low recovery of pAmpCs can possibly be explained by using a ligation library preparation kit, which has a lower recovery of plasmids compared to a rapid detection kit (Wick et al., 2021). For ESBLs, specificity was lower at 70%. Several ß-lactamases were misclassified on the ONT platform, such as the broad-spectrum ß-lactamase TEM-1 as ESBL TEM-10, or the broad-spectrum ß-lactamases SHV-1, SHV-11, or inhibitor-resistant broad-spectrum ß-lactamase SHV-26 as ESBLs SHV-12 or SHV-152. The lower fidelity on the allele level for ONT—due to lower quality sequencing data—has been previously observed (Tamma et al., 2019).

The proposed workflow to predict relevant clinical phenotypes (Figure 1) had multiple decision gateways. Early misclassification propagated downstream through the pipeline. More specifically, if an isolate was incorrectly identified on the first grouping level, this error propagated to the final classification. As an example, the phenotypically third-generation cephalosporin-resistant, ESBL-positive isolate CRE596KLPN is classified as such using Illumina sequencing data, but on the ONT platform, ertapenem resistance is overcalled in the absence of a carbapenemase gene. Hence, this isolate is classified as non-CP CRE using ONT due to carbapenem classification occurring upstream of third-generation cephalosporin classification. Nevertheless, the ESBL genes are also identified in this isolate on the ONT platform. As a mitigation strategy, we placed the WGS-AST models at the beginning of the decision workflow, since we have shown that those models have a higher error tolerance than simple AMR marker detection. This allows generally to compensate for the lower sensitivity of pAmpCs and lower specificity of ESBLs on ONT sequencing data, which is more often the case than vice versa. Co-detection of AMR genes together with WGS-AST is important for tailoring therapy as this discrimination enables selection of the correct downstream treatment more precisely.

The higher performance of ONT compared to Illumina was observed for antimicrobials where identification of markers or marker classes is sufficient for resistance calling. This was, e.g., the case for ceftriaxone and amikacin, where the presence of CTX-M class markers can be sufficient to predict ceftriaxone resistance, or amikacin which is dependent on ribosomal RNA methyltransferases such as *armA* or *rmtA* homologs (Bonnet, 2004; Galimand et al., 2012; Liakopoulos et al., 2016). The lower performance of individual antimicrobials on the ONT platform was more often observed when the resolution of SNPs is crucial for resistance calling, as it is, e.g., the case for *gyrA* and *parC* mutations for fluoroquinolones, or mutations in efflux pumps for piperacillin-tazobactam, or when detection of multiple aminoglycoside-modifying enzymes is required, as it is the case for gentamicin resistance (Chen et al., 2003; Fu et al., 2013).

There are several limitations to this study. First, some species were represented by a relatively small number (*E. coli* [n: 14], *E. cloacae* [n: 7]) of isolates within the 181 clinical isolates. As some AMR markers are specific to individual bacterial species, this work needs to be repeated on a larger sample set. Second, all isolates came from a single site (geographic region) resulting in likely over- or under-representation of individual AMR markers based on the genetic relatedness of isolates. This work is currently in the proof-of-concept stage and should be expanded in the future to include geographically diverse isolates. Third, the Phred quality score of ONT is still inferior to Illumina, although it has increased by an order of magnitude since the beginning of the study, both by new sequencing chemistries and due to improved bioinformatics. WGS-AST models and related bioinformatic pipelines need to be adapted accordingly.

With the availability of sequencing platforms in hospital practice, risk identification based on AMR classes can enhance patient management. For the routine application of WGS-AST, clinical microbiology laboratories need to implement automated approaches for DNA extraction, library preparation, and sequencing, such as fully automated clinical microbial cultivation and identification systems. Once established, methodological control of WGS-AST might be easier to maintain compared to BMD-AST (e.g., daily internal controls) and skills/requirements for routine use might be lower. Additionally, WGS-AST allows drawing retrospective conclusions about antimicrobials that were not available at the time of sequencing, for example, for AST surveillance studies.

The performance gap for WGS-AST between ONT and Illumina has narrowed over the last years, especially with the upcoming of newer base calling models, flow cells, and sequencing chemistries of higher accuracy, which routinely generate reads at a modal raw read accuracy at a Phred score of 20, which is an improvement from an accuracy of 90–95% (10 < Phred <13) to 99% (Phred = 20). However, Illumina sequencing reads typically achieve Phred scores of above 30 (> 99.9% accuracy). Having assemblies of good quality is important for WGS-AST predictions, genotyping, and identification of AMR classes if single-nucleotide polymorphisms are involved. While significant differences still exist, especially for the detection of AMR genes, our results highlight the potential of the ONT platform for consideration in clinical microbiology laboratories if combined with robust bioinformatics methodology. Combining AMR genotypes and WGS-AST predictions can help guide effective therapeutic management decisions.

## Data availability statement

The data presented in the study are deposited in the NCBI Sequence Read Archive (SRA) repository, accession number PRJNA825705.

## Author contributions

RC: formal analysis, investigation, data curation, software, validation, writing—original draft, writing—reviewing and editing, and visualization. YB, SL, and EJ: data curation, formal analysis, and writing—reviewing and editing. PM: data curation, software, investigation, and validation. SB: software, methodology, data curation, writing—reviewing and editing, supervision, and project administration. PT: conceptualization and writing—reviewing and editing. PS: conceptualization, methodology, writing—reviewing and editing, and supervision. All authors contributed to the article and approved the submitted version.

## Conflict of interest

RC, PM, and SB are employees of Ares Genetics GmbH. PS has received grants and personal fees from Accelerate Diagnostics, OpGen Inc., and BD Diagnostics; grants from bioMérieux, Inc., Affinity Biosensors, and Hardy Diagnostics; and personal fees from Roche Diagnostics, GeneCapture, and Shionogi Inc.

The remaining authors declare that the research was conducted in the absence of any commercial or financial relationships that could be construed as a potential conflict of interest.

## Publisher’s note

All claims expressed in this article are solely those of the authors and do not necessarily represent those of their affiliated organizations, or those of the publisher, the editors and the reviewers. Any product that may be evaluated in this article, or claim that may be made by its manufacturer, is not guaranteed or endorsed by the publisher.

## Supplementary material

SUPPLEMENTARY TABLE 1 List of the species-antimicrobial pairs used for the WGS-AST models.

SUPPLEMENTARY TABLE 2 Summary of BMD-AST results. n: number of evaluated isolates.

SUPPLEMENTARY TABLE 3 Average Nucleotide Identity (ANI) analysis applied to the samples with discrepant species identification results.

SUPPLEMENTARY TABLE 4 List of all AMR markers identified in the Illumina and ONT assemblies.

SUPPLEMENTARY TABLE 5 Performance metrics for all species-antimicrobial pairs by sequencing platform. CA: categorical agreement, VME: very major error, ME: major error, TP: true positive, FP: false positive, FN: false negative, TN: true negative, n: number of evaluated species-antimicrobial pairs.

SUPPLEMENTARY TABLE 6 Performance metrics of WGS-AST on Illumina and ONT assemblies, broken down by taxon. CA: categorical agreement, VME: very major error, ME: major error, TP: true positive, FP: false positive, FN: false negative, TN: true negative, n: number of evaluated species-antimicrobial pairs.

SUPPLEMENTARY TABLE 7 Performance metrics of WGS-AST on Illumina and ONT assemblies, broken down by antimicrobial. CA: categorical agreement, VME: very major error, ME: major error, TP: true positive, FP: false positive, FN: false negative, TN: true negative, n: number of evaluated species-antimicrobial pairs.

SUPPLEMENTARY TABLE 8 WGS-AST performance metrics of ertapenem broken down by BMD-AST phenotype for carbapenem. CA: categorical agreement, VME: very major error, ME: major error, TP: true positive, FP: false positive, FN: false negative, TN: true negative, n: number of evaluated species-antimicrobial pairs.

SUPPLEMENTARY TABLE 9 List of all isolates from the study with their species identification, relevant identified markers, WGS-AST for ertapenem and ceftriaxone, and the resulting predicted phenotype group.
